# *N*′-(Adamantan-2-yl­idene)thio­phene-2-carbohydrazide

**DOI:** 10.1107/S1600536811044758

**Published:** 2011-10-29

**Authors:** Adnan A. Kadi, Amer M. Alanzi, Ali A. El-Emam, Seik Weng Ng, Edward R. T. Tiekink

**Affiliations:** a Department of Pharmaceutical Chemistry, College of Pharmacy, King Saud University, Riyadh, Saudi Arabia; b Department of Chemistry, University of Malaya, 50603 Kuala Lumpur, Malaysia; c Chemistry Department, Faculty of Science, King Abdulaziz University, PO Box 80203 Jeddah, Saudi Arabia

## Abstract

In the title mol­ecule, C_15_H_18_N_2_OS, a small twist is noted, with the dihedral angle between the central carbohydrazone residue (r.m.s. deviation = 0.029 Å) and the thio­phene ring being 12.47 (10)°. The *syn* arrangement of the amide H and carbonyl O atoms allows for the formation of centrosymmetric dimers *via* N—H⋯O hydrogen bonds. These are linked in the three-dimensional structure by C—H⋯π inter­actions. The thio­phene ring is disordered over two co-planar orientations, the major component having a site-occupancy factor of 0.833 (2).

## Related literature

For the biological activity of adamantane derivatives see: Vernier *et al.* (1969 ▶); El-Emam *et al.* (2004 ▶). For background to our work into the biological activity of adamantane derivatives, see: Kadi *et al.* (2010 ▶); Al-Omar *et al.* (2010 ▶). For a related structure, see: Al-Tamimi *et al.* (2010 ▶).
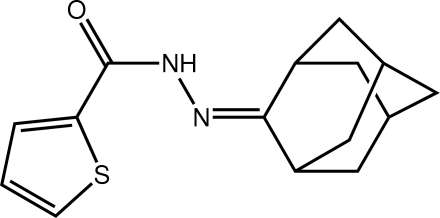


## Experimental

### 

#### Crystal data

C_15_H_18_N_2_OS
*M* *_r_* = 274.37
Monoclinic, 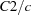

*a* = 16.7262 (2) Å
*b* = 12.5663 (1) Å
*c* = 13.5562 (2) Å
β = 102.473 (1)°
*V* = 2782.08 (6) Å^3^
*Z* = 8
Cu *K*α radiation
μ = 2.01 mm^−1^
*T* = 100 K
0.30 × 0.25 × 0.20 mm

#### Data collection

Agilent SuperNova Dual diffractometer with an Atlas detector
Absorption correction: multi-scan (*CrysAlis PRO*; Agilent, 2010 ▶) *T*_min_ = 0.584, *T*_max_ = 0.690
5687 measured reflections
2849 independent reflections
2671 reflections with *I* > 2σ(*I*)
*R*_int_ = 0.015

#### Refinement

*R*[*F* ^2^ > 2σ(*F* ^2^)] = 0.032
*wR*(*F* ^2^) = 0.087
*S* = 1.04
2849 reflections
189 parameters
10 restraints
H atoms treated by a mixture of independent and constrained refinement
Δρ_max_ = 0.38 e Å^−3^
Δρ_min_ = −0.39 e Å^−3^

### 

Data collection: *CrysAlis PRO* (Agilent, 2010 ▶); cell refinement: *CrysAlis PRO*; data reduction: *CrysAlis PRO*; program(s) used to solve structure: *SHELXS97* (Sheldrick, 2008 ▶); program(s) used to refine structure: *SHELXL97* (Sheldrick, 2008 ▶); molecular graphics: *ORTEP-3* (Farrugia, 1997 ▶) and *DIAMOND* (Brandenburg, 2006 ▶); software used to prepare material for publication: *publCIF* (Westrip, 2010 ▶).

## Supplementary Material

Crystal structure: contains datablock(s) global, I. DOI: 10.1107/S1600536811044758/hg5127sup1.cif

Structure factors: contains datablock(s) I. DOI: 10.1107/S1600536811044758/hg5127Isup2.hkl

Supplementary material file. DOI: 10.1107/S1600536811044758/hg5127Isup3.cml

Additional supplementary materials:  crystallographic information; 3D view; checkCIF report

## Figures and Tables

**Table 1:** Hydrogen-bond geometry (Å, °) *Cg*1 is the centroid of the S1,C1–C4 ring.

*D*—H⋯*A*	*D*—H	H⋯*A*	*D*⋯*A*	*D*—H⋯*A*
N1—H1⋯O1^i^	0.93 (2)	1.92 (2)	2.844 (1)	173 (2)
C13—H13⋯*Cg*1^ii^	1.00	2.61	3.5791 (16)	163
C15—H15a⋯*Cg*1^iii^	0.99	2.69	3.5683 (16)	148

Symmetry codes: (i) 
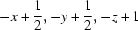
; (ii) 
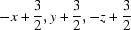
; (iii) 
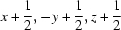
.

**Fig. 1.**
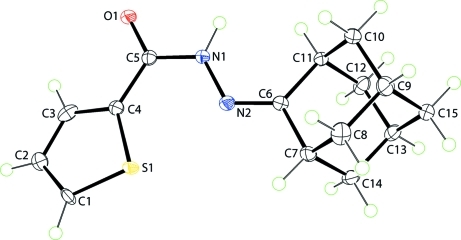
The molecular structure of (I) showing the atom-labelling scheme and displacement ellipsoids at the 50% probability level. Only the major component of the disordered thiophene ring is shown for reasons of clarity.

**Fig. 2.**
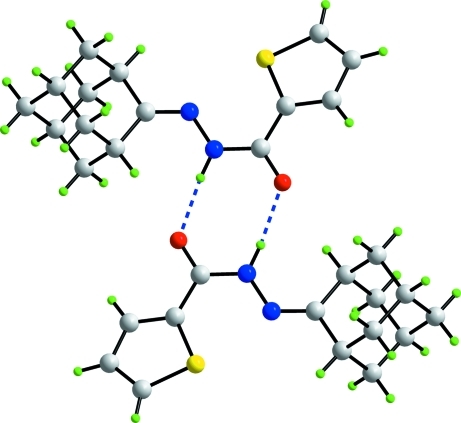
Centrosymmetric dimer in (I) sustained by N—H···O hydrogen bonds shown as blue dashed lines.

**Fig. 3.**
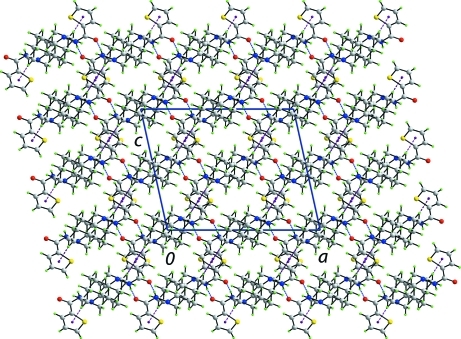
Unit-cell contents for (I) shown in projection down the b axis. The N—H···O hydrogen bonds and C—H···π interactions are shown as blue and purple dashed lines, respectively.

## supplementary crystallographic information

### Comment

Derivatives of adamantane have long been known for their diverse biological activities including anti-viral activity against the influenza (Vernier *et al.*, 1969) and HIV viruses (El-Emam *et al.*, 2004). In continuation of our interest in the chemical and pharmacological properties of adamantane derivatives (Kadi *et al.*, 2010; Al-Omar *et al.*, 2010; Al-Tamimi *et al.*, 2010), we synthesized the title compound, *N*'-(2-thienylcarbonyl)-2-adamantanone hydrazone, (I), as a potential chemotherapeutic agent. Herein, the crystal and molecular structures are described.

A small twist is noted in the molecule of (I), Fig. 1, as seen in the value of the dihedral between the thiophene ring and the central carbohydrazone residue (O1,N1,N2,C4,C5; r.m.s. deviation = 0.029 Å) being 12.47 (10)°. In the major component of the disordered molecule, the thiophene-S atom is proximate to the hydrazone-N atom, S1···N1 = 2.7797 (11) Å, whereas in the minor component, the C3'—H3' atom is 2.29 Å from N2. The amide-H and carbonyl-O atoms are *syn*. This arrangement allows for the formation of centrosymmetric dimers, Fig. 2 and Table 1, and eight-membered {···HNCO}_2_ synthons. The dimers thus formed are consolidated in the crystal packing by C—H···π interactions, Fig. 3 and Table 1.

### Experimental

Thiophene-2-carbohydrazide (1.42 g, 0.01 mol) and 2-adamantanone (1.5 g, 0.01 mol) were heated in ethanol (10 ml) for 4 h. The solid that separated upon cooling was collected and recrystallized from ethanol to yield 2.44 g (98%) of C_15_H_18_N_2_OS as colourless crystals, *M*.pt. 464–467 K. The formulation was established by solution NMR spectroscopy. ^1^H-NMR (CDCl_3_): δ 1.91–2.05 (*m*, 14 adamantyl-H), 7.12 (*s*, 1 thienyl H), 7.60–7.63 (*m*, 1 thienyl H), 8.17 (*s*, 1 thienyl H), 10.04 (s, 1 amino H) p.p.m.. ^13^C-NMR: 27.78, 30.86, 36.38, 37.81, 39.21, 163.32 (adamantyl), 129.25, 133.78,134.30, 134.90 (thienyl)), 164.15 (carbonyl) p.p.m..

### Refinement

Carbon-bound H-atoms were placed in calculated positions [C—H 0.95 to 1.00 Å, *U*_iso_(H) 1.2–1.5*U*_eq_(C)] and were included in the refinement in the riding model approximation. The amino H-atom was located in a difference Fourier map, and was freely refined. The thienyl ring was disordered over two positions in respect of the sulfur atom and three of the four carbon atoms; the carbon atom connected to the carbonyl group is ordered. The C—S single bond distances were restrained to 1.71±0.01 Å, the formal C—C double-bond distances were restrained to 1.36±0.01 Å and the formal C—C single-bond distances to 1.46±0.01 Å. The anisotropic displacement parameters of the primed atoms were set to those of the unprimed ones. The major component refined to a site occupancy factor = 0.833 (1).

### Crystal data

**Table:**

C_15_H_18_N_2_OS	*F*(000) = 1168
*M**_r_* = 274.37	*D*_x_ = 1.310 Mg m^−^^3^
Monoclinic, *C*2/*c*	Cu *K*α radiation, λ = 1.54184 Å
Hall symbol: -C 2yc	Cell parameters from 4116 reflections
*a* = 16.7262 (2) Å	θ = 3.3–76.3°
*b* = 12.5663 (1) Å	µ = 2.01 mm^−^^1^
*c* = 13.5562 (2) Å	*T* = 100 K
β = 102.473 (1)°	Prism, colourless
*V* = 2782.08 (6) Å^3^	0.30 × 0.25 × 0.20 mm
*Z* = 8	

### Data collection

**Table:**

Agilent SuperNova Dual diffractometer with an Atlas detector	2849 independent reflections
Radiation source: SuperNova (Cu) X-ray Source	2671 reflections with *I* > 2σ(*I*)
Mirror	*R*_int_ = 0.015
Detector resolution: 10.4041 pixels mm^-1^	θ_max_ = 76.5°, θ_min_ = 4.4°
ω scan	*h* = −20→21
Absorption correction: multi-scan (*CrysAlis PRO*; Agilent, 2010)	*k* = −15→15
*T*_min_ = 0.584, *T*_max_ = 0.690	*l* = −16→11
5687 measured reflections	

### Refinement

**Table:**

Refinement on *F*^2^	Primary atom site location: structure-invariant direct methods
Least-squares matrix: full	Secondary atom site location: difference Fourier map
*R*[*F*^2^ > 2σ(*F*^2^)] = 0.032	Hydrogen site location: inferred from neighbouring sites
*wR*(*F*^2^) = 0.087	H atoms treated by a mixture of independent and constrained refinement
*S* = 1.04	*w* = 1/[σ^2^(*F*_o_^2^) + (0.0511*P*)^2^ + 1.8544*P*] where *P* = (*F*_o_^2^ + 2*F*_c_^2^)/3
2849 reflections	(Δ/σ)_max_ = 0.001
189 parameters	Δρ_max_ = 0.38 e Å^−^^3^
10 restraints	Δρ_min_ = −0.39 e Å^−^^3^

### Fractional atomic coordinates and isotropic or equivalent isotropic displacement parameters (Å^2^)

**Table:**

	*x*	*y*	*z*	*U*_iso_*/*U*_eq_	Occ. (<1)
S1	0.32529 (2)	0.46310 (4)	0.24150 (3)	0.01360 (14)	0.833 (2)
C1	0.26341 (15)	0.55387 (19)	0.1659 (2)	0.0172 (5)	0.833 (2)
H1A	0.2777	0.5884	0.1098	0.021*	0.833 (2)
C2	0.1909 (2)	0.5709 (3)	0.1958 (2)	0.0187 (4)	0.833 (2)
H2	0.1493	0.6191	0.1646	0.022*	0.833 (2)
C3	0.18762 (10)	0.5073 (2)	0.2781 (2)	0.0223 (5)	0.833 (2)
H3	0.1417	0.5081	0.3088	0.027*	0.833 (2)
S1'	0.16790 (19)	0.5096 (3)	0.2790 (2)	0.01360 (14)	0.167 (2)
C1'	0.2016 (12)	0.5693 (15)	0.1820 (12)	0.0172 (5)	0.167 (2)
H1'	0.1703	0.6204	0.1384	0.021*	0.167 (2)
C2'	0.2765 (11)	0.5375 (13)	0.1731 (14)	0.0187 (4)	0.167 (2)
H2'	0.3044	0.5617	0.1234	0.022*	0.167 (2)
C3'	0.3071 (7)	0.4624 (12)	0.2493 (10)	0.0223 (5)	0.167 (2)
H3'	0.3588	0.4285	0.2565	0.027*	0.167 (2)
O1	0.19394 (5)	0.34953 (7)	0.42862 (7)	0.0180 (2)	
N1	0.32838 (6)	0.32219 (8)	0.44331 (8)	0.0143 (2)	
H1	0.3254 (10)	0.2681 (13)	0.4887 (12)	0.023 (4)*	
N2	0.39858 (6)	0.33902 (8)	0.40601 (7)	0.0147 (2)	
C4	0.25352 (7)	0.44346 (9)	0.31283 (8)	0.0135 (2)	
C5	0.25708 (7)	0.36818 (9)	0.39748 (9)	0.0137 (2)	
C6	0.46677 (7)	0.30469 (9)	0.45855 (9)	0.0137 (2)	
C7	0.54146 (7)	0.31718 (10)	0.41432 (9)	0.0172 (3)	
H7	0.5274	0.3575	0.3493	0.021*	
C8	0.57170 (8)	0.20454 (11)	0.39590 (10)	0.0216 (3)	
H8A	0.5290	0.1666	0.3464	0.026*	
H8B	0.6214	0.2095	0.3676	0.026*	
C9	0.59118 (7)	0.14246 (10)	0.49565 (10)	0.0194 (3)	
H9	0.6107	0.0694	0.4835	0.023*	
C10	0.51402 (7)	0.13459 (10)	0.53943 (10)	0.0195 (3)	
H10A	0.5265	0.0941	0.6035	0.023*	
H10B	0.4706	0.0962	0.4914	0.023*	
C11	0.48369 (7)	0.24706 (10)	0.55881 (9)	0.0147 (2)	
H11	0.4329	0.2428	0.5862	0.018*	
C12	0.55174 (7)	0.30665 (11)	0.63301 (9)	0.0193 (3)	
H12A	0.5328	0.3792	0.6449	0.023*	
H12B	0.5644	0.2687	0.6985	0.023*	
C13	0.62905 (7)	0.31363 (10)	0.58972 (9)	0.0177 (3)	
H13	0.6731	0.3514	0.6388	0.021*	
C14	0.60877 (7)	0.37598 (10)	0.49038 (10)	0.0196 (3)	
H14A	0.6585	0.3830	0.4623	0.024*	
H14B	0.5897	0.4483	0.5028	0.024*	
C15	0.65806 (7)	0.20126 (10)	0.57098 (9)	0.0184 (3)	
H15A	0.6712	0.1615	0.6355	0.022*	
H15B	0.7084	0.2056	0.5439	0.022*	

### Atomic displacement parameters (Å^2^)

**Table:**

	*U*^11^	*U*^22^	*U*^33^	*U*^12^	*U*^13^	*U*^23^
S1	0.0146 (3)	0.0164 (2)	0.0113 (2)	−0.00024 (18)	0.00607 (15)	0.00223 (12)
C1	0.0211 (13)	0.0172 (11)	0.0118 (8)	0.0019 (8)	0.0004 (8)	0.0070 (7)
C2	0.0190 (12)	0.0181 (7)	0.0184 (12)	0.0026 (7)	0.0026 (6)	0.0013 (8)
C3	0.0147 (11)	0.0256 (8)	0.0287 (8)	−0.0040 (10)	0.0096 (9)	−0.0030 (7)
S1'	0.0146 (3)	0.0164 (2)	0.0113 (2)	−0.00024 (18)	0.00607 (15)	0.00223 (12)
C1'	0.0211 (13)	0.0172 (11)	0.0118 (8)	0.0019 (8)	0.0004 (8)	0.0070 (7)
C2'	0.0190 (12)	0.0181 (7)	0.0184 (12)	0.0026 (7)	0.0026 (6)	0.0013 (8)
C3'	0.0147 (11)	0.0256 (8)	0.0287 (8)	−0.0040 (10)	0.0096 (9)	−0.0030 (7)
O1	0.0134 (4)	0.0194 (4)	0.0217 (4)	0.0002 (3)	0.0053 (3)	0.0062 (3)
N1	0.0125 (5)	0.0153 (5)	0.0153 (5)	0.0001 (4)	0.0036 (4)	0.0035 (4)
N2	0.0139 (5)	0.0153 (5)	0.0159 (5)	0.0005 (4)	0.0052 (4)	0.0016 (4)
C4	0.0149 (5)	0.0128 (5)	0.0125 (5)	−0.0014 (4)	0.0024 (4)	−0.0010 (4)
C5	0.0141 (5)	0.0118 (5)	0.0147 (5)	−0.0010 (4)	0.0021 (4)	−0.0015 (4)
C6	0.0149 (5)	0.0128 (5)	0.0138 (5)	0.0000 (4)	0.0041 (4)	0.0006 (4)
C7	0.0160 (6)	0.0213 (6)	0.0160 (6)	0.0026 (4)	0.0068 (4)	0.0059 (5)
C8	0.0214 (6)	0.0266 (7)	0.0176 (6)	0.0041 (5)	0.0062 (5)	−0.0034 (5)
C9	0.0187 (6)	0.0145 (6)	0.0249 (6)	0.0031 (4)	0.0045 (5)	0.0001 (5)
C10	0.0167 (6)	0.0152 (6)	0.0251 (6)	−0.0025 (4)	0.0009 (5)	0.0059 (5)
C11	0.0115 (5)	0.0189 (6)	0.0141 (5)	−0.0009 (4)	0.0035 (4)	0.0040 (4)
C12	0.0155 (6)	0.0274 (7)	0.0148 (6)	−0.0014 (5)	0.0029 (5)	−0.0027 (5)
C13	0.0130 (5)	0.0202 (6)	0.0196 (6)	−0.0035 (4)	0.0028 (4)	−0.0011 (5)
C14	0.0153 (5)	0.0161 (6)	0.0289 (7)	−0.0009 (4)	0.0081 (5)	0.0051 (5)
C15	0.0132 (5)	0.0204 (6)	0.0215 (6)	0.0012 (4)	0.0037 (5)	0.0060 (5)

### Geometric parameters (Å, °)

**Table:**

S1—C4	1.7142 (12)	C7—C8	1.5415 (18)
S1—C1	1.7215 (15)	C7—C14	1.5412 (17)
C1—C2	1.377 (2)	C7—H7	1.0000
C1—H1A	0.9500	C8—C9	1.5340 (18)
C2—C3	1.382 (3)	C8—H8A	0.9900
C2—H2	0.9500	C8—H8B	0.9900
C3—C4	1.362 (2)	C9—C15	1.5319 (17)
C3—H3	0.9500	C9—C10	1.5358 (17)
S1'—C4	1.634 (3)	C9—H9	1.0000
S1'—C1'	1.712 (9)	C10—C11	1.5432 (17)
C1'—C2'	1.344 (9)	C10—H10A	0.9900
C1'—H1'	0.9500	C10—H10B	0.9900
C2'—C3'	1.411 (10)	C11—C12	1.5406 (16)
C2'—H2'	0.9500	C11—H11	1.0000
C3'—C4	1.391 (8)	C12—C13	1.5336 (16)
C3'—H3'	0.9500	C12—H12A	0.9900
O1—C5	1.2414 (14)	C12—H12B	0.9900
N1—C5	1.3499 (15)	C13—C14	1.5313 (17)
N1—N2	1.3912 (13)	C13—C15	1.5321 (17)
N1—H1	0.926 (17)	C13—H13	1.0000
N2—C6	1.2820 (15)	C14—H14A	0.9900
C4—C5	1.4784 (16)	C14—H14B	0.9900
C6—C7	1.5062 (15)	C15—H15A	0.9900
C6—C11	1.5118 (15)	C15—H15B	0.9900
			
C4—S1—C1	91.53 (11)	C7—C8—H8A	109.7
C2—C1—S1	112.5 (3)	C9—C8—H8B	109.7
C2—C1—H1A	123.7	C7—C8—H8B	109.7
S1—C1—H1A	123.7	H8A—C8—H8B	108.2
C1—C2—C3	109.8 (3)	C15—C9—C8	109.14 (10)
C1—C2—H2	125.1	C15—C9—C10	109.09 (10)
C3—C2—H2	125.1	C8—C9—C10	109.70 (10)
C4—C3—C2	116.73 (19)	C15—C9—H9	109.6
C4—C3—H3	121.6	C8—C9—H9	109.6
C2—C3—H3	121.6	C10—C9—H9	109.6
C4—S1'—C1'	91.4 (7)	C9—C10—C11	109.97 (10)
C2'—C1'—S1'	114.0 (15)	C9—C10—H10A	109.7
C2'—C1'—H1'	123.0	C11—C10—H10A	109.7
S1'—C1'—H1'	123.0	C9—C10—H10B	109.7
C1'—C2'—C3'	109.4 (17)	C11—C10—H10B	109.7
C1'—C2'—H2'	125.3	H10A—C10—H10B	108.2
C3'—C2'—H2'	125.3	C6—C11—C12	108.82 (10)
C4—C3'—C2'	112.8 (12)	C6—C11—C10	106.87 (10)
C4—C3'—H3'	123.6	C12—C11—C10	109.43 (10)
C2'—C3'—H3'	123.6	C6—C11—H11	110.5
C5—N1—N2	119.91 (10)	C12—C11—H11	110.5
C5—N1—H1	116.8 (10)	C10—C11—H11	110.5
N2—N1—H1	121.6 (10)	C13—C12—C11	110.07 (10)
C6—N2—N1	117.79 (10)	C13—C12—H12A	109.6
C3—C4—C3'	105.5 (6)	C11—C12—H12A	109.6
C3—C4—C5	122.63 (13)	C13—C12—H12B	109.6
C3'—C4—C5	131.8 (6)	C11—C12—H12B	109.6
C3'—C4—S1'	112.5 (6)	H12A—C12—H12B	108.2
C5—C4—S1'	115.42 (13)	C14—C13—C15	110.10 (10)
C3—C4—S1	109.44 (12)	C14—C13—C12	108.72 (10)
C5—C4—S1	127.90 (9)	C15—C13—C12	109.52 (10)
S1'—C4—S1	116.54 (13)	C14—C13—H13	109.5
O1—C5—N1	119.64 (10)	C15—C13—H13	109.5
O1—C5—C4	119.35 (10)	C12—C13—H13	109.5
N1—C5—C4	120.98 (10)	C13—C14—C7	109.52 (10)
N2—C6—C7	117.30 (10)	C13—C14—H14A	109.8
N2—C6—C11	129.20 (11)	C7—C14—H14A	109.8
C7—C6—C11	113.42 (9)	C13—C14—H14B	109.8
C6—C7—C8	107.35 (10)	C7—C14—H14B	109.8
C6—C7—C14	109.42 (10)	H14A—C14—H14B	108.2
C8—C7—C14	109.31 (10)	C13—C15—C9	110.08 (10)
C6—C7—H7	110.2	C13—C15—H15A	109.6
C8—C7—H7	110.2	C9—C15—H15A	109.6
C14—C7—H7	110.2	C13—C15—H15B	109.6
C9—C8—C7	109.81 (10)	C9—C15—H15B	109.6
C9—C8—H8A	109.7	H15A—C15—H15B	108.2
			
C4—S1—C1—C2	1.5 (2)	S1—C4—C5—N1	15.62 (17)
S1—C1—C2—C3	−1.4 (3)	N1—N2—C6—C7	176.23 (10)
C1—C2—C3—C4	0.4 (4)	N1—N2—C6—C11	−0.37 (18)
C4—S1'—C1'—C2'	1.7 (16)	N2—C6—C7—C8	−115.56 (12)
S1'—C1'—C2'—C3'	−1(2)	C11—C6—C7—C8	61.57 (13)
C1'—C2'—C3'—C4	−1(2)	N2—C6—C7—C14	125.92 (11)
C5—N1—N2—C6	171.74 (11)	C11—C6—C7—C14	−56.95 (13)
C2—C3—C4—C3'	−0.8 (7)	C6—C7—C8—C9	−58.56 (13)
C2—C3—C4—C5	−177.5 (2)	C14—C7—C8—C9	60.04 (13)
C2—C3—C4—S1'	−161 (2)	C7—C8—C9—C15	−60.03 (13)
C2—C3—C4—S1	0.8 (3)	C7—C8—C9—C10	59.45 (13)
C2'—C3'—C4—C3	−0.4 (14)	C15—C9—C10—C11	59.76 (12)
C2'—C3'—C4—C5	175.8 (10)	C8—C9—C10—C11	−59.75 (13)
C2'—C3'—C4—S1'	2.4 (16)	N2—C6—C11—C12	−126.71 (13)
C2'—C3'—C4—S1	−160 (10)	C7—C6—C11—C12	56.58 (13)
C1'—S1'—C4—C3	18.8 (18)	N2—C6—C11—C10	115.21 (13)
C1'—S1'—C4—C3'	−2.3 (11)	C7—C6—C11—C10	−61.49 (12)
C1'—S1'—C4—C5	−176.8 (7)	C9—C10—C11—C6	58.68 (12)
C1'—S1'—C4—S1	−0.8 (7)	C9—C10—C11—C12	−58.99 (13)
C1—S1—C4—C3	−1.28 (18)	C6—C11—C12—C13	−57.90 (13)
C1—S1—C4—C3'	19 (9)	C10—C11—C12—C13	58.55 (13)
C1—S1—C4—C5	176.83 (14)	C11—C12—C13—C14	61.27 (13)
C1—S1—C4—S1'	1.40 (18)	C11—C12—C13—C15	−59.07 (13)
N2—N1—C5—O1	177.15 (10)	C15—C13—C14—C7	59.00 (12)
N2—N1—C5—C4	−4.97 (16)	C12—C13—C14—C7	−60.97 (13)
C3—C4—C5—O1	11.4 (2)	C6—C7—C14—C13	58.13 (13)
C3'—C4—C5—O1	−164.3 (9)	C8—C7—C14—C13	−59.16 (13)
S1'—C4—C5—O1	9.0 (2)	C14—C13—C15—C9	−59.43 (12)
S1—C4—C5—O1	−166.49 (9)	C12—C13—C15—C9	60.06 (13)
C3—C4—C5—N1	−166.50 (17)	C8—C9—C15—C13	59.55 (13)
C3'—C4—C5—N1	17.8 (9)	C10—C9—C15—C13	−60.30 (13)
S1'—C4—C5—N1	−168.90 (17)		

### Hydrogen-bond geometry (Å, °)

**Table:**

Cg1 is the centroid of the S1,C1–C4 ring.

**Table:**

*D*—H···*A*	*D*—H	H···*A*	*D*···*A*	*D*—H···*A*
N1—H1···O1^i^	0.93 (2)	1.92 (2)	2.844 (1)	173 (2)
C13—H13···Cg1^ii^	1.00	2.61	3.5791 (16)	163
C15—H15a···Cg1^iii^	0.99	2.69	3.5683 (16)	148

Symmetry codes: (i) −*x*+1/2, −*y*+1/2, −*z*+1; (ii) −*x*+3/2, *y*+3/2, −*z*+3/2; (iii) *x*+1/2, −*y*+1/2, *z*+1/2.
